# Supplementary material for: Sensitivity of point-of-care testing C reactive protein and procalcitonin to diagnose urinary tract infections in Dutch nursing homes: PROGRESS study protocol
Source: BMJ Open. 2019 Aug 10;9(8):e031269. doi: 10.1136/bmjopen-2019-031269 (PMC6701568; doi:10.1136/bmjopen-2019-031269)
Supplement: Author's manuscript [file bmjopen-2019-031269.draft_revisions.pdf]

BMJ Open is committed to open peer review. As part of this commitment we make the peer review history of every article we publish publicly available.

When an article is published we post the peer reviewers' comments and the authors' responses online. We also post the versions of the paper that were used during peer review. These are the versions that the peer review comments apply to.

The versions of the paper that follow are the versions that were submitted during the peer review process. They are not the versions of record or the final published versions. They should not be cited or distributed as the published version of this manuscript.

BMJ Open is an open access journal and the full, final, typeset and author-corrected version of record of the manuscript is available on our site with no access controls, subscription charges or pay-per-view fees (http://bmjopen.bmj.com).

If you have any questions on BMJ Open's open peer review process please email info.bmjopen@bmj.com

BMJ Open

Point of care testing of C-reactive protein and procalcitonin to diagnose urinary tract infections in nursing homes: PROGRESS study protocol

Journal:	*BMJ Open*
Manuscript ID	bmjopen-2019-031269
Article Type:	Protocol
Date Submitted by the Author:	25-Apr-2019
Complete List of Authors:	Kuil, Sacha; Amsterdam UMC, Medical Microbiology;<br>Hidad, Soemeja; Amsterdam UMC, Medical Microbiology<br>Fischer, Johan; Amsterdam UMC, Clinical Chemistry<br>Harting, Janneke; Amsterdam UMC, Public Health<br>Hertogh, C; VU University Medical Center , General Practice and Elderly Care Medicine<br>Prins, Jan; Amsterdam UMC, Internal Medicine<br>van Leth, Frank; AIGHD, Global Health<br>de Jong, Menno; Amsterdam UMC, Medical Microbiology<br>Schneeberger, Caroline; Amsterdam UMC, Medical Microbiology
Keywords:	Point of care testing, C-Reactive protein, Procalcitonin, Nursing homes, Urinary tract infections < UROLOGY, Antimicrobial resistance

SCHOLARONE™
Manuscripts

Point of care testing of C-reactive protein and procalcitonin to diagnose urinary tract infections in nursing homes: PROGRESS study protocol

S.D. Kuil[1#] S. Hidad[1] J.C. Fischer[2] J. Harting[3] C.M.P.M. Hertogh[4] J.M. Prins[5] F. van Leth[6, 7] M.D. de Jong[1] C. Schneeberger[1]

[1] s.d.kuil@amc.nl Amsterdam UMC, University of Amsterdam, Department of Medical Microbiology, Amsterdam Infection & Immunity Institute, Meibergdreef 9, Amsterdam, The Netherlands

[1] s.hidad@amc.nl Amsterdam UMC, University of Amsterdam, Department of Medical Microbiology, Amsterdam Infection & Immunity Institute, Meibergdreef 9, Amsterdam, The Netherlands

[2] j.c.fischer@amc.nl Amsterdam UMC, University of Amsterdam, Department of Clinical Chemistry, Meibergdreef 9, Amsterdam, The Netherlands

[3] j.harting@amc.nl Amsterdam UMC, University of Amsterdam, Department of Public Health, Amsterdam Public Health research institute, Meibergdreef 9, Amsterdam, The Netherlands

[4] cmpm.hertogh@vumc.nl Amsterdam UMC, VU University Medical Center, Department of General Practice and Elderly Care Medicine, Amsterdam Public Health, University Network of Organizations for Elderly Care, de Boelelaan 1117, Amsterdam, The Netherlands.

[5] j.m.prins@amc.nl Amsterdam UMC, University of Amsterdam, Division of Infectious Diseases, Department of Internal Medicine, Amsterdam Infection & Immunity Institute, Meibergdreef 9, Amsterdam, The Netherlands

[6] f.vanleth@aighd.org Amsterdam UMC, University of Amsterdam , Department of Global Health, Meibergdreef 9, Amsterdam, The Netherlands

[7] Amsterdam Institute for Global Health and Development, Amsterdam, The Netherlands.

[1] m.d.dejong@amc.nl Amsterdam UMC, University of Amsterdam, Department of Medical Microbiology, Amsterdam Infection & Immunity Institute, Meibergdreef 9, Amsterdam, The Netherlands

[1] c.schneeberger@amc.nl Amsterdam UMC, University of Amsterdam, Department of Medical Microbiology, Amsterdam Infection & Immunity Institute, Meibergdreef 9, Amsterdam, The Netherlands

[#] Corresponding author: Sacha D. Kuil, s.d.kuil@amc.nl, Dept. of Medical Microbiology Amsterdam UMC, University of Amsterdam, Meibergdreef 9, 1105 AZ Amsterdam, the Netherlands

Word count abstract: 304 **Word count full text:** 4166

For peer review only - http://bmjopen.bmj.com/site/about/guidelines.xhtml

ABSTRACT

Introduction:

Suspected urinary tract infection (UTI) ranks among the most common reasons for antibiotic use in nursing homes. However, diagnosing UTI in this setting is challenging because UTI often present with non-specific symptomatology. Moreover asymptomatic bacteriuria is common in elderly, which complicates attribution of causality to detection of bacteria in urine. These diagnostic challenges contribute to overuse of antibiotics and emergence of antimicrobial resistance (AMR) in nursing homes. Given the diagnostic challenges there is a need for point-of-care (POC) diagnostic tests to support clinical rules for diagnosing UTI. Procalcitonin (PCT) and C-reactive protein (CRP) are inflammatory blood markers that have been proven useful to support diagnosis and monitoring of (bacterial) respiratory tract infections and sepsis. While limited studies suggest their usefulness in supporting UTI diagnosis, their utility has not been studied in elderly populations for this purpose.

Methods and analysis:

In an 24-month matched diagnostic accuracy study 'PROGRESS' will assess and compare the sensitivity of rapid POC measurements of blood CRP and PCT levels to support clinical rules for diagnosing UTI in nursing home residents. The primary outcome measure is sensitivity of the POC tests to identify patients with true UTI based on the predefined definition, as derived from Receiving Operating Curves (ROC).

Discussion:

This study will show the sensitivity of CRP and PCT in the diagnostic process of a UTI in elderly. When effective, the potential impact of CRP and/or PCT measured by POC diagnostic tests on antibiotic prescription needs to be established in a consecutive study.

Ethics and dissemination:

This study will be conducted in accordance with Good Clinical Practice guidelines and the principles of the Declaration of Helsinki. The study protocol is approved by the Medical Ethical Committee (METc) of AmsterdamUMC location VUmc with reference number 2017.350 and National Central Committee on Research involving Human Subjects (CCMO) with reference number NL62067.029.17.

Registration details: Dutch trial registry: NTR6467

For peer review only - http://bmjopen.bmj.com/site/about/guidelines.xhtml

Keywords: Point of care testing, C-Reactive protein, Procalcitonin, Nursing homes, Urinary tract infections, Antimicrobial resistance

Strengths and limitations

Strengths

- Stringent post-hoc UTI criteria incorporating microbiology result and clinical response to adequate antibiotic therapy
- Diagnostic accuracy study in relevant study population, namely nursing home residents

Limitations

- No on-site POCT, however POCT is performed within 4 hours
- Single country study

Protocol version 4 March 2019, version 5

1 September 2017 Original

21 October 2017 Amendment 1: Addition of information brochures for nursing home staff and patients

20 November 2017 Amendment 2: Change of nomenclature for part of nursing home residents to temporary rehabilitation patients (at rehabilitation wards)

7 March 2018 Amendment 3: Change exclusion criterion prior inclusion to prior inclusion in the past 30 days. Subsequently to account for non-independence of observation for a limited number of participants, the sample size is adjusted with a design effect of 1.1

28 June 2018 Amendment 4: (1) Change in blood sample collection method (venipuncture to finger prick collection) due to availability of new procalcitonin POCT with small input volume, as finger prick blood sample collection is a preferred collection method in the elderly nursing home population. (2) Change sample size retaining stringent criteria (p-value 0.05 and power 90%) (3) Prolongation study duration: 12 to 18 months (4) Expansion of number of nursing homes from 11 to 12 nursing homes

3 March 2019 Amendement 5: (1) Expansion of number of nursing homes from 12 to 13 nursing homes (2) Addition of extra urine test for detection of gram-negative bacteria (3) Adjusted informed consent procedure for capacitated residents

For peer review only - http://bmjopen.bmj.com/site/about/guidelines.xhtml

For peer review only

Introduction

Antimicrobial resistance (AMR) is mainly driven by inappropriate antibiotic use in both humans and animals. AMR is an problem worldwide. Nursing homes are increasingly regarded as an important reservoir for the emergence of AMR[1 2 3 4].

The most frequently reported infections in elderly residents are urinary tract infections (UTI)[5]. In Dutch nursing homes, an average weekly incidence of 10.3 (95% CI 9.8 – 10.8) per 1000 elderly residents was found[6]. UTI is the most common reason for prescribing antibiotics in Dutch nursing homes. However, not all diagnosed UTIs are “true” UTIs since recognition and diagnosing UTI in nursing homes is complex. Approximately one third of elderly residents UTIs are misdiagnosed, leading to inappropriate antibiotic use[7 8]. This is mainly due to the facts that non-specific, non-urinary tract symptoms such as altered mental status are often attributed to UTIs whilst asymptomatic bacteriuria (ASB), possibly resulting in positive urine tests, is also common in the elderly residents.[9]

Cognitive impairments and urinary tract infections

The majority of nursing homes in the Netherlands consist of psychogeriatric wards (57%), for elderly residents suffering from cognitive impairments, mainly Alzheimer disease[10 1112 13]. Their ability to verbally communicate or express classical symptoms of UTI, such as dysuria, urgency or frequency, is often limited[14]. The most frequently presented symptom in elderly residents leading to antibiotic prescription for a suspected UTI is an altered mental status (43.3%), while classical symptoms as dysuria, urgency and frequency are present in the minority of cases (0 – 3.8%)[15]. Confusion or an altered mental state are nonspecific symptoms and can result from other infectious and non-infectious diseases in the elderly residents.

Asymptomatic bacteriuria

Asymptomatic bacteriuria (ASB) is defined by the presence of significant bacteriuria without symptoms of UTI. ASB is thus regarded as colonization of the urinary tract rather than infection. ASB is highly prevalent in healthy elderly persons with reported prevalence rates as high as 40-50%[16 17]. In the presence of ASB, frequently used urine tests based on detection of bacteria are less applicable to diagnose UTI, because detection of bacteria does not discriminate between

For peer review only - http://bmjopen.bmj.com/site/about/guidelines.xhtml

ASB and UTI. In combination with above described non-specific symptomatology, it is difficult to distinguish ASB from “true” UTI in the elderly.

In conclusion, decisions about who to treat and who not to treat are challenging in elderly residents with suspected UTI, resulting in potential antibiotic overuse in those who do not need treatment, Availability of a simple and rapid point-of-care (POC) test to distinguish true UTI from ASB represents an unmet need which would greatly assist clinical management of these vulnerable patients, not only by improving appropriateness of antibiotic treatment but also by enabling consideration of alternative causes of presenting non-specific symptomatology when UTI is excluded.

C-reactive protein and procalcitonin

In the diagnosis of respiratory tract infections and monitoring of bacterial sepsis, inflammatory markers such as C-reactive protein (CRP) and procalcitonin (PCT) in blood have proven useful to guide antibiotic therapy and reduce antibiotic use[18 19]. Currently, point-of-care (POC) CRP measurements are recommended by Dutch guidelines for general practitioners to guide antibiotic treatment for acute respiratory tract infections. CRP and PCT represent potential candidates for rapid POC testing to support UTI diagnosis.

Ageing and frailty are associated with changes in serum inflammation protein levels, such as CRP and PCT [20 21 22]. Therefore, it is important to determine cut-off values for CRP and PCT specifically in the elderly nursing home resident population.

Inflammatory markers CRP and PCT in UTI

Studies in adults showed that CRP and PCT levels in parenchymatous infections (acute pyelonephritis, prostatitis and epididymitis) are increased[23 24]. Using a PCT-based algorithm in UTI treatment was shown to reduce antibiotic exposure in adults[25]. In a subgroup analysis including elderly (> 70 years of age) with lower UTI antibiotic exposure was reduced as well, suggesting a potential role for PCT.

A study in children showed that CRP or PCT can help to distinguish renal scarring due to a UTI from renal scarring due to another reason and between pyelonephritis (upper UTI) and cystitis (lower UTI)[26 27 28]. However studies are small or were performed retrospectively. The specificity

For peer review only - http://bmjopen.bmj.com/site/about/guidelines.xhtml

and positive predictive values were low because CRP and PCT were also increased in children with lower UTI. This suggests a possible role for inflammatory markers in distinguishing a UTI (local inflammation of the bladder) from no inflammation.

In the elderly population only two studies have been performed evaluating CRP and PCT and their possible role in UTI[29 30]. However these two studies focused on hospitalized and more severely ill elderly populations. Therefore these results are not applicable to the nursing home population. However, in 13% of the healthy elderly controls CRP values were increased, again suggesting increased inflammation in ageing and frailty, indicating the need to determine specific cutoffs in elderly. Studies on lower UTIs and CRP or PCT in elderly are scarce.

Distinguishing UTI and ASB

The majority of UTIs in elderly residents are lower UTIs without fever or other signs of systemic illness[31 32] while most studies on CRP and PCT in UTI focus on diagnostic values in upper UTI or focus on distinguishing upper and lower UTI. The only small study on distinguishing lower UTI (infection) from ASB (colonization) in adults, shows a high negative predictive value (NPV) for UTI of PCT levels at a cutoff of 0.25 ng/mL[33] , suggesting that low PCT levels can rule out UTI and contribute to reducing antibiotic use.

Methods and analysis

Aim

To assess the utility of point-of-care measurements of blood CRP and PCT levels to support clinical rules for diagnosing urinary tract infections UTI in elderly nursing home residents.

Outcome

The primary outcome is the sensitivity of the point-of-care (POC) test to identify patients with a true UTI based on the predefined definition, as derived from Receiving Operating Curves (ROC).

Design and setting

In a prospective matched diagnostic accuracy study we will assess and compare the sensitivity

For peer review only - http://bmjopen.bmj.com/site/about/guidelines.xhtml

of rapid POC measurements of blood CRP and PCT levels to support clinical rules for diagnosing UTI in elderly residents, with a post-hoc definition of UTI with stringent criteria including microbiology results as gold standard.

In this study a true UTI is present when the following five criteria are met: presence of at least two urinary or non-specific symptoms (1), positive urine leucocyte esterase test (2), presence of uropathogens in bacterial culture at $10^4 \geq$ CFU/mL (3), maximum of two uropathogens present (4) and symptom resolution in the course of adequate antibiotic treatment, where adequate treatment is defined by proven susceptibility of isolated uropathogens to the administered antibiotic (5).

The matching refers to the assessment of blood CRP and PCT levels in the same study participants. The study will be performed in nursing homes of the University Network for Organizations of Elderly Care of the VUmc University Medical Center (UNO-VUmc). The study duration is 18 months.

The nursing home population in this study consists mostly of psychogeriatric and somatic (long-stay) wards and some rehabilitation (short-stay) wards.

Informed consent procedure

Most nursing home patients are incapacitated. In case of incapacity legal representatives will be asked for informed consent. Capacitated patients will be asked for informed consent themselves. When nursing home staff suspect a UTI, it is not considered practical to obtain written informed consent from the representatives because preferably the blood sample should be drawn as soon as possible. Therefore informed consent will be obtained pre-emptively at the start of the study or when admitted to the nursing home. This means that patients or their legal representatives provide consent a priori to participate in the study once a UTI is clinically suspected during the study period. This procedure will greatly enhance feasibility of enrolment in psychogeriatric nursing home wards.

For peer review only - http://bmjopen.bmj.com/site/about/guidelines.xhtml

Inclusion and exclusion criteria

Eligible for study participation are elderly nursing home residents clinically suspected of UTI by the attending physician or nurse. Exclusion criteria are suspected respiratory tract infection, suspected other infection requiring antibiotic therapy, previous study inclusion in the past 30 days or lack of written informed consent.

Study procedures

Study enrolment

The study physician will be notified by the nursing home staff when there is potentially eligible patient and will visits the nursing home as soon as possible. The research physician will verify if eligibility criteria are met and will complete study enrolment.

Data collection

To meet our post-hoc UTI criteria data on signs and symptoms, type and duration of antibiotic used, urine leucocyte esterase, bacterial culture and antimicrobial susceptibility and clinical response are collected. Demographic, clinical and laboratory data are collected through an electronic data capture system using software of Open Data Kit[34] with which case report forms (CRFs) are designed that incorporate consistency checks to minimize incompatible data points. Data will be handled encoded, working with barcodes scanned by the database APP. Data of paper registration forms is directly entered into online CRFs and uploaded to a pre-defined database.

Data on culture results (species, susceptibility patterns by Minimal Inhibitory Concentrations MIC's) from the laboratory system will be collected in the currently used laboratory systems (Labtrain and Kiestra)

Data on signs and symptoms, clinical response and antibiotic use

Demographic and clinical data are collected at the day of study enrolment by the attending physician or nurse.

The research physician will visit the nursing homes regularly for monitoring of follow-up. The attending physician or nurse will evaluate clinical response at day 5 and 10 after study enrolment. Improvement of clinical symptoms, compared to symptoms at enrolment is evaluated. Data on antibiotic use (timing of initial antimicrobial therapy, type of antimicrobial

For peer review only - http://bmjopen.bmj.com/site/about/guidelines.xhtml

agent and possible switch in antimicrobial therapy) will be collected by the research physician 10 days after study enrolment.

Urine sample collection, dipstick analysis and bacterial culture

The attending nurse will collect a urine sample for purpose of this study. Urine samples are collected spontaneously voiding either directly in a sterile urine container or in chamber pot (insert pan). When participants have an indwelling urine catheter, urine will be collected when the urine bag is changed. However when participants suffer from urinary incontinence or are not able to urinate on the toilet or chamber pot, diapers will be used to obtain urine for bacterial culture and dipstick analysis [35 36] Urine extraction from diapers can provide a reliable diagnostic specimen, if fecal contamination can reasonably excluded[37]. Date, time and way of urine collection method are registered by the attending nurse. The urine sample is stored at $4^{o}C$. For dipstick analysis Combur 2 (Roche Diagnostics) will be used (nitrite and leucocyte esterase). Urines will be used for semi-quantitative bacterial culture. Inoculation of 10 μL urine to CHROMID CPS Elite agar (Biomerieux) and Columbia CNA agar with 5% sheep blood (Biomerieux) will be streaked using a four quadrant pattern. Bacterial growth will be interpreted after overnight incubation at $35^{o}C$ in aerobic (CPSE) and CO_2 enriched (CNA) environment. Uropathogens will be identified by Maldi-tof mass spectrometry (Microflex, Bruker Daltonic). When bacterial growth of $\geq 10^4$ CFU/mL is found, antibiotic susceptibility testing will be performed using the VITEK2 platform (BioMérieux).

Blood sample collection and point of care testing

For POC testing the research physician will collect a blood sample by capillary fingerprick. Capillary blood sample collection is a preferred collection method in the elderly nursing home population. For CRP testing the Afinion AS100 point-of-care (POC) platform (Alere Health B.V., Tilburg) is used. For PCT testing the Afias1 PCT Plus (Avant Medical B.V., Geffen) is used. Both, CRP and PCT platforms enable capillary blood sample collection to better facilitate future implementation in nursing homes. POC testing are performed according to the manufacturer's protocol. For logistic reasons the POC testing is performed centrally at the Medical Microbiology laboratory of our hospital. POC tests are performed within 4 hours after blood sample collection to ensure stability of CRP and PCT.

Participants, attending physicians and nurses will not be informed of POC-test and urinary

For peer review only

For peer review only - http://bmjopen.bmj.com/site/about/guidelines.xhtml

culture results.

Biological specimen storage and molecular analysis In future

Aliquots of urine samples will be stored (-80°C) when consent is obtained specifically for genetic studies. In future studies metagenomic sequencing will be performed on bacterial DNA, to identify uropathogens and resistance genes.

Sample size

For the sample size calculation, we used a two-sided p-value of 0.05 and a power of 90%. We assume a difference in sensitivity between the two POC tests of at least 10% as clinically relevant. This difference is the net result of discordant test outcomes between the two tests. The magnitude of discordance is the main parameter in the McNemar test used to calculate the sample size for the matched design.

With a proposed sample size of 440 enrolled participants, we are able to adequately assess an increased sensitivity of 10% or more, when the prevalence of UTI in the study population is 40% or more. If the prevalence if UTI is lower, the difference in prevalence that is statistically significant (at the 5%) level increases to 11 or 12%.

Based on data of the national sentinel surveillance network for infectious diseases in nursing homes (SNIV) in 2015, the prevalence of UTI in Dutch nursing homes is 10.3 in 1000 patient weeks. UTI is defined by SNIV as patients with nonspecific or urinary tract complaints and a positive result on nitrite or leucocyte esterase in dipstick analysis and an urine culture positive for a uropathogen.

In order to reach the target, the PROGRESS study will take place in 13 nursing homes with around 1350 elderly residents in total. Recruitment of a manageable number of approximately 12 participants per week in total is anticipated.

Data and statistical analysis

The sensitivity of both POC tests to diagnose UTIs defined by the post-hoc definition is derived from the optimal point of a Receiver Operating Curve (ROC). Sensitivities of CRP and PCT tests are compared by a matched analysis approach using a two-tailed McNemar test assessing the statistical significance of differences between the sensitivities of each pair of tests (performed in

For peer review only - http://bmjopen.bmj.com/site/about/guidelines.xhtml

each study participant).

This study protocol is written using the SPIRIT reporting guidelines[38]

Ethics and dissemination:

This study will be conducted in accordance with Good Clinical Practice guidelines and the principles of the Declaration of Helsinki. The study protocol is approved by the Medical Ethical Committee (METc) of AmsterdamUMC location VUmc with reference number 2017.350 and National Central Committee on Research involving Human Subjects (CCMO) with reference number NL62067.029.17. Written informed consent from nursing home residents or legal representatives (when incapacitated) will be obtained prior to study enrolment. Informed consent from residents will be obtained directly by researchers or via mail. Informed consent from legal representatives will be obtained via mail. Treating physicians decide which residents are capacitated.

Data safety monitoring board

No data safety monitoring board has been appointed for this study as the risks of this study are assumed negligible.

Discussion

To our knowledge there is no other (ongoing) trial evaluating the diagnostic value of C-reactive protein and procalcitonin in urinary tract infections in elderly[39]. This trial will hopefully provide evidence for supporting the diagnosis of UTI in elderly by increased levels of inflammatory markers, to guide diagnosis and treatment and aiming at reduction of antibiotic prescription rates and resistance rates in nursing homes. Since effectiveness of a new test does not just depend on its proven efficacy in research studies, but also on successful implementation after the study, qualitative research on identifying the barriers and facilitators for implementation are needed and will be performed in parallel to the described study. The potential impact of CRP and/or PCT measured by POC diagnostic tests on antibiotic prescription for UTI in elderly nursing home residents needs to be established in a consecutive study once evidence of their utility for diagnosing UTI has been obtained from the current planned study.

For peer review only - http://bmjopen.bmj.com/site/about/guidelines.xhtml

List of abbreviations

Amsterdam UMC Amsterdam Universitair Medische Centra
AMR Antimicrobial resistance
ASB Asymptomatic bacteriuria
CCMO National Central Committee on Research involving Human Subjects
CFU Colony forming units
CRP C-reactive protein
LQAS Lot Quality Assurance Sampling
MIC Minimal inhibitory concentration
NPV Negative predictive value
ODK Open data kit
PCT Procalcitonin
POC Point of care
SNIV Surveillance network for infectious diseases in nursing homes
UNO Universitair netwerk ouderenzorg
UTI urinary tract infection
VUmc Vrije Universiteit medisch centrum

Declarations

Dissemination policy

According to the CCMO statement on publication policy, the results of this study will be disclosed unreservedly.

Competing interests

This study is funded by The Netherlands Organization for Health Research and Development (ZonMW).

Funding

This study is funded by The Netherlands Organization for Health Research and Development

For peer review only - http://bmjopen.bmj.com/site/about/guidelines.xhtml

(ZonMW) grant 541001003. ZonMW, Laan van Nieuw Oost Indië 334, 2593 CE Den Haag, The Netherlands. The study protocol is being peer reviewed by the funder. The funder has no role in collection, management, analysis, and interpretation of data, writing of the report or the decision to submit the report for publication.

Roles and responsibilities

Research physician/PhD student (SDK): coordinating PROGRESS, patient recruiting, data collection, specimen handling, POC and urine testing, preparation protocols, CRFs and publication of study reports including annual ethical committee report.

Research assistant (SH): patient recruiting, data collection, specimen handling, POC and urine testing

Principal investigator (MdJ), senior researcher (CS) and lead epidemiologist (FvL): Study planning, agreement of final protocols, reviewing progress of study

Lead epidemiologist (FvL): design of the electronic report form (eCRF), database design and maintenance, data validation and data management plans, user account maintenance, design of automated data validation scripts, preparation of the final data anlysis sets, supervising of data analysis.

Laboratory chemist (JCF): responsible for verification of POCT, contractual issues with manufacturers

In each participating center a lead investigator (elderly care physician) will be identified, to be responsible for progress monitoring and assisting with study set-up per site.

Author contributions

SDK recruits patients, coordinates and performs the clinical study and the wrote the manuscript. SH recruits patients and performs the clinical study. FvL, JCF, JH, NM, CMPMH, JMP, MDdJ and CS developed the protocol and secured funding for this project. CS, FvL and MdJ supervised the design of the study and writing this manuscript. FvL provided the statistical analysis plan, database set-up. All authors have read and approved the manuscript.

Patient and public involvement

Patients or public are not involved in designing this study.

For peer review only

For peer review only

For peer review only - http://bmjopen.bmj.com/site/about/guidelines.xhtml

437 **References**

[1] Rooney PJ, O'Leary MC, Loughrey AC, McCalmont M, Smyth B, Donaghy P, Badri M, Woodford N, Karisik E, Livermoore DM Nursing homes as a reservoir of extended-spectrum beta-lactamase (ESBL)-producing ciprofloxacin-resistant Escherichia coli. J Antimicrob Chemother. 2009;64(3):635-41.
[2] Lee BY, Bartsch SM, Wong KF, Singh A, Avery TL, Kim DS, Brown ST, Murphey CR, Yilmaz SL, Potter MA, Huang SS. The importance of nursing homes in the spread of methicillin-resistant Staphylococcus aureus (MRSA) among hospitals. Med Care. 2013;51(3):205-15.
[3] Kahvecioglu D, Ramiah K, McMaughan D, Garfinkel S, McSorley VE, Nguyen QN, Yang M, Pugliese C, Mehr D, Philips CD. Multidrug-resistant organism infections in US nursing homes: a national study of prevalence, onset, and transmission across care settings, October 1, 2010-December 31, 2011. Infect Control Hosp Epidemiol. 2014;35 Suppl 3:S48-55.
[4] Bedenic B, Beader N, Godic-Torkar K, Vranić-Ladavac M, Luxner J, Veir Z, Grisold AJ, Zarfel G. Nursing Home as a Reservoir of Carbapenem-Resistant Acinetobacter baumannii. Microb Drug Resist. 2015;21(3):270-8.
[5] Lutters M, Vogt-Ferrier NB. Antibiotic duration for treating uncomplicated, symptomatic lower urinary tract infections in elderly women. Cochrane Database of Systematic Reviews. 2008; doi:10.1002/14651858.CD001535.pub2
[6] Rijksinstituut voor Volksgezondheid en Milieu. Surveilance Netwerk Infectieziekten in Verpleeghuizen. Resultaten van wekelijkse surveillance. Referentiecijfers 2011 – 2015 https://www.rivm.nl/sites/default/files/2018-11/Referentiecijfers%20Incidentie%20SNIV%202011-2015_def.pdf Accessed 7 March 2019
[7] van Buul LW, Veenhuizen RB, Achterberg WP, Schellevis FG, Essink RT, de Greeff SC, Natsch S, van der Steen JT, Hertogh CM. Antibiotic Prescribing In Dutch Nursing Homes: How Appropriate Is It? J Am Med Dir Assoc. 2015; 16(3):229-37
[8] Loeb M, Simor AE, Landry L, et al. Antibiotic use in Ontario facilities that provide chronic care. J Gen Intern Med. 2001;16:376–383.
[9] van Buul LW, Vreeken HL, Bradley SF, Crnich CJ, Drinka PJ, Geerlings SE, Jump RLP, Mody L, Mylotte JJ, Loeb M, Nace DA, Nicolle LE, Sloane PD, Stuart RL, Sundvall PD, Ulleryd P, Veenhuizen RB, Hertogh CMPM. The Development of a Decision Tool for the Empiric Treatment of Suspected Urinary Tract Infection in Frail Older Adults: A Delphi Consensus Procedure. J Am Med Dir Assoc. 2018. 19(9):757-764
[10] Schols, J and Kardol T. Dementia care in nursing homes requires a multidisciplinary approach. In: Schüssler S and Lohrmann C. Dementia in nursing homes. 2017. Chapter 15, p. 210.
[11] Alzheimer's disease facts and figures. Alzheimer's Association. 2017 Alzheimer's & Dementia 2017;13(4):325-373
[12] Shah DC, Evans M, King D. Prevalence of mental illness in a rehabilitation unit for older adults. Postgrad Med J. 2000; 76(893):153-6.
[13] Heeren THJ, Lagaay AM, Rooijmans HGM. De prevalentie van het dementiesyndroom bij de oudste bewoners van het somatisch verpleeghuis. Ned Tijdsch Geneeskd 1992;136(14):695-698
[14] Sobel JD and Kaye D. Urinary tract infections. In: Mandell, Douglas and Bennett. Principles and practice of infectious diseases 8th edition, Chapter 74, p.896
[15] D'Agata, E, Loeb MB, and Mitchell SL. Challenges Assessing Nursing Home Residents with Advanced Dementia for Suspected Urinary Tract Infections. J Am Geriatr Soc. 2013;61(1):62–66
[16] Hedin K, Petersson C, Widebäck K, Kahlmeter G, Mölstad S. Asymptomatic bacteriuria in a population of elderly in municipal institution care. Scand J Prim Health Care. 2002;20(3):166-8
[17] Eberle CM, Winsemius D, Garibaldi RA. Risk factors and consequences of bacteriuria in non catheterized nursing home residents. J Geront 1993;48(6):M266-71
[18] Do NT, Ta NT, Tran NT, et al. Point-of-care C-reactive protein testing to reduce inappropriate use of antibiotics for non-severe acute respiratory infections in Vietnamese primary health care: a randomised controlled trial. Lancet Glob Health. 2016;4(9):e633-41
[19] Agency for Health Care Research and Quality. Procalcitonine-guided antibiotic therapy. Comparative Effectiveness Review. 2012; (78):1-16

For peer review only - http://bmjopen.bmj.com/site/about/guidelines.xhtml

https://effectivehealthcare.ahrq.gov/sites/default/files/related_files/procalcitonin_executive.pdf Accessed 7 March 2019
[20] Soysal P, Stubbs B, Lucato P, Luchini C, Solmi M, Peluso R, Sergi G, Isik AT, Manzato E, Maggi S, Maggio M, Prina AM, Cosco TD, Wu YT, Veronese N. Inflammation and frailty in the elderly: A systematic review and meta-analysis. Ageing Research Reviews. 2016;(31):1-8
[21] Chenevier-Gobeaux C, Trabattoni E, Elfassy Y, Picard C, Guérin S, Borderie D, Claessens YE. Decisional procalcitonin thresholds are not adapted to elderly patients admitted to the emergency room. Biomarkers. 2012;17(5):477-481.
[22] Woloshin S, Schwartz LM Distribution of C-reactive protein values in the United States. N Engl J Med 2005;352(15):1611-3.
[23] Sugimoto K, Shimizu N, Matsumura N, Oki T, Nose K, Nishioka T, Uemura H. Procalcitonin as a useful marker to decide upon intervention for urinary tract infection. Infect Drug Resist. 2013;7(6):83-6.
[24] Chuang YC, Vikas T, Liu RT, Chancellor MB, Tyagi P. Urine and Serum C-Reactive Protein Levels as Potential Biomarkers of Lower Urinary Tract Symptoms. Urol Sci 2010;21(3):132-136.
[25] Drozdov D, Schwarz S, Kutz A, Grolimund E, Rast AC, Steiner D, Regez K, Schild U, Guglielmetti M, Conca A, Reutlinger B, Ottinger C, Buchkremer F, Haubitz S, Blum, C, Huber A, Buergi U, Schuetz P, Bock A, Fux CA, Mueller B, Albrich WC. Procalcitonin and pyuria-based algorithm reduces antibiotic use in urinary tract infections: a randomized controlled trial. BMC Med. 2015;1;13:104.
[26] Yildiz B, Poyraz H, Cetin N, Kural N, Colak O. High sensitive C-reactive protein: a new marker for urinary tract infection, VUR and renal scar. Eur Rev Med Pharmacol Sci. 2013;17(19):2598-604
[27] Pecile P, Miorin E, Romanello C, Falleti E, Valent F, Giacomuzzi F, Tenore A. Procalcitonin: A Marker of Severity of Acute Pyelonephritis Among Children Pediatrics. 2009;114(2):e249-54.
[28] Xu RY, Liu HW, Liu JL, Dong JH. Procalcitonin and C-reactive protein in urinary tract infection diagnosis. BMC Urol. 2014;30:14-45.
[29] Lai CC, Chen SY, Wang CY, Wang JY, Su CP, Liao CH, Tan CK, Huang YT, Lin HI, Hsueh PR. Diagnostic Value of Procalcitonin for Bacterial Infection in Elderly Patients in the Emergency Department J Am Geriatr Soc. 2010;58(3):518-22
[30] Dwolatzky T, Olshtain-Pops K, Yinnon AM, Raveh D, Rogowski O, Shapira I, Rotstein R, Berliner S, Rudensky B. Procalcitonin in the elderly: normal plasma concentrations and response to bacterial infections. Eur J Clin Microbiol Infect Dis. 2005;24(11):763-5.
[31] Johansen TE1, Botto H, Cek M, Grabe M, Tenke P, Wagenlehner FM, Naber KG. Critical review of current definitions of urinary tract infections and proposal of an EAU/ESIU classification system. Int J Antimicrob Agents. 2011;38 Suppl:64-70
[32] Foxman B. Urinary tract infection syndromes: occurrence, recurrence, bacteriology, risk factors, and disease burden. Infect Dis Clin North Am. 2014; 28(1):1-13.
[33] Levine AR, Tran M, Shepherd J, Naut E. Utility of initial procalcitonin values to predict urinary tract infection. American Journal of Emergency Medicine. 2018; 36(11):1993-1997.
[34] Open Data Kit http://www.opendatakit.org/ Accessed 7 March 2019
[35] Midthun SJ, Paur RA, Lindseth G, Von Duvillard SP.Bacteriuria detection with a urine dipstick applied to incontinence pads of nursing home residents. Geriatr Nurs. 2003;24(4):206-9
[36] Belmin J, Hervias Y, Avellano E, Oudart O, Durand I. Reliability of sampling urine from disposable diapers in elderly incontinent women. J Am Geriatr Soc. 1993;41(11):1182-6
[37] Diaper urine: a reliable alternative for obtaining urine samples for UTI diagnosis in elderly suffering from urine incontinence. Poster session P2137, 24 April 2018 Session: Urinary tract infection - risks, diagnosis, treatment. ECCMID 2018 Madrid, Spain. https://www.escmid.org/escmid_publications/escmid_elibrary/ Accessed 7 March 2019
[38] Chan A-W, Tetzlaff JM, Altman DG, Laupacis A, Gøtzsche PC, Krleža-Jerić K, Hróbjartsson A, Mann H, Dickersin K, Berlin J, Doré C, Parulekar W, Summerskill W, Groves T, Schulz K, Sox H, Rockhold FW, Rennie D, Moher D. SPIRIT 2013 Statement: Defining standard protocol items for clinical trials. Ann Intern Med. 2013;158(3):200-207
[39] ISRCTN registry http://controlled-trials.com Accessed 7 March 2019

For peer review only - http://bmjopen.bmj.com/site/about/guidelines.xhtml

Reporting checklist for protocol of a clinical trial.

Based on the SPIRIT guidelines.

Instructions to authors

Complete this checklist by entering the page numbers from your manuscript where readers will find each of the items listed below.

Your article may not currently address all the items on the checklist. Please modify your text to include the missing information. If you are certain that an item does not apply, please write "n/a" and provide a short explanation.

Upload your completed checklist as an extra file when you submit to a journal.

In your methods section, say that you used the SPIRIT reporting guidelines, and cite them as:

Chan A-W, Tetzlaff JM, Altman DG, Laupacis A, Gøtzsche PC, Krleža-Jerić K, Hróbjartsson A, Mann H, Dickersin K, Berlin J, Doré C, Parulekar W, Summerskill W, Groves T, Schulz K, Sox H, Rockhold FW, Rennie D, Moher D. SPIRIT 2013 Statement: Defining standard protocol items for clinical trials. Ann Intern Med. 2013;158(3):200-207

		Reporting Item	Page Number
Title	#1	Descriptive title identifying the study design, population, interventions, and, if applicable, trial acronym	1
Trial registration	#2a	Trial identifier and registry name. If not yet registered, name of intended registry	2
Trial registration: data set	#2b	All items from the World Health Organization Trial Registration Data Set https://www.who.int/ictrp/network/trds_1.2.1/en/	First enrollment: 23-11-2017. Recruitment status: recruiting<br><br>Other items in manuscript
Protocol version	#3	Date and version identifier	3
Funding	#4	Sources and types of financial, material, and other support	12

For peer review only

Roles and responsibilities: contributorship	#5a	Names, affiliations, and roles of protocol contributors	13
Roles and responsibilities: sponsor contact information	#5b	Name and contact information for the trial sponsor<br><br>Contact information medical microbiologie AmsterdamUMC: Prof. Dr. M.D. de Jong University of Amsterdam, Department of Medical Microbiology, Amsterdam Infection & Immunity Institute, Meibergdreef 9, Amsterdam, The Netherlands	12
Roles and responsibilities: sponsor and funder	#5c	Role of study sponsor and funders, if any, in study design; collection, management, analysis, and interpretation of data; writing of the report; and the decision to submit the report for publication, including whether they will have ultimate authority over any of these activities	12, 13
Roles and responsibilities: committees	#5d	Composition, roles, and responsibilities of the coordinating centre, steering committee, endpoint adjudication committee, data management team, and other individuals or groups overseeing the trial, if applicable (see Item 21a for data monitoring committee)	13
Background and rationale	#6a	Description of research question and justification for undertaking the trial, including summary of relevant studies (published and unpublished) examining benefits and harms for each intervention	4-6
Background and rationale: choice of comparators	#6b	Explanation for choice of comparators	4-6
Objectives	#7	Specific objectives or hypotheses	6
Trial design	#8	Description of trial design including type of trial (eg, parallel group, crossover, factorial, single group), allocation ratio, and framework (eg, superiority, equivalence, non-inferiority, exploratory)	6, 7

For peer review only

Study setting	#9	Description of study settings (eg, community clinic, academic hospital) and list of countries where data will be collected. Reference to where list of study sites can be obtained	6, 7
Eligibility criteria	#10	Inclusion and exclusion criteria for participants. If applicable, eligibility criteria for study centres and individuals who will perform the interventions (eg, surgeons, psychotherapists)	8
Interventions: description	#11a	Interventions for each group with sufficient detail to allow replication, including how and when they will be administered	n/a observational study
Interventions: modifications	#11b	Criteria for discontinuing or modifying allocated interventions for a given trial participant (eg, drug dose change in response to harms, participant request, or improving / worsening disease)	n/a observational study
Interventions: adherance	#11c	Strategies to improve adherence to intervention protocols, and any procedures for monitoring adherence (eg, drug tablet return; laboratory tests)	n/a observational study
Interventions: concomitant care	#11d	Relevant concomitant care and interventions that are permitted or prohibited during the trial	n/a
Outcomes	#12	Primary, secondary, and other outcomes, including the specific measurement variable (eg, systolic blood pressure), analysis metric (eg, change from baseline, final value, time to event), method of aggregation (eg, median, proportion), and time point for each outcome. Explanation of the clinical relevance of chosen efficacy and harm outcomes is strongly recommended	6
Participant timeline	#13	Time schedule of enrolment, interventions (including any run-ins and washouts), assessments, and visits for participants. A schematic diagram is highly recommended (see Figure)	8, 9
Sample size	#14	Estimated number of participants needed to achieve study objectives and how it was determined, including	10

For peer review only

		clinical and statistical assumptions supporting any sample size calculations	
Recruitment	#15	Strategies for achieving adequate participant enrolment to reach target sample size	10
Allocation: sequence generation	#16a	Method of generating the allocation sequence (eg, computer-generated random numbers), and list of any factors for stratification. To reduce predictability of a random sequence, details of any planned restriction (eg, blocking) should be provided in a separate document that is unavailable to those who enrol participants or assign interventions	n/a observational study
Allocation concealment mechanism	#16b	Mechanism of implementing the allocation sequence (eg, central telephone; sequentially numbered, opaque, sealed envelopes), describing any steps to conceal the sequence until interventions are assigned	n/a observational study
Allocation: implementation	#16c	Who will generate the allocation sequence, who will enrol participants, and who will assign participants to interventions	n/a
Blinding (masking)	#17a	Who will be blinded after assignment to interventions (eg, trial participants, care providers, outcome assessors, data analysts), and how	n/a observational study
Blinding (masking): emergency unblinding	#17b	If blinded, circumstances under which unblinding is permissible, and procedure for revealing a participant's allocated intervention during the trial	n/a observational study
Data collection plan	#18a	Plans for assessment and collection of outcome, baseline, and other trial data, including any related processes to promote data quality (eg, duplicate measurements, training of assessors) and a description of study instruments (eg, questionnaires, laboratory tests) along with their reliability and validity, if known. Reference to where data collection forms can be found, if not in the protocol	8
Data collection plan: retention	#18b	Plans to promote participant retention and complete follow-up, including list of any outcome data to be	8

For peer review only

		collected for participants who discontinue or deviate from intervention protocols	
Data management	#19	Plans for data entry, coding, security, and storage, including any related processes to promote data quality (eg, double data entry; range checks for data values). Reference to where details of data management procedures can be found, if not in the protocol	8
Statistics: outcomes	#20a	Statistical methods for analysing primary and secondary outcomes. Reference to where other details of the statistical analysis plan can be found, if not in the protocol	10
Statistics: additional analyses	#20b	Methods for any additional analyses (eg, subgroup and adjusted analyses)	n/a
Statistics: analysis population and missing data	#20c	Definition of analysis population relating to protocol non-adherence (eg, as randomised analysis), and any statistical methods to handle missing data (eg, multiple imputation)	10
Data monitoring: formal committee	#21a	Composition of data monitoring committee (DMC); summary of its role and reporting structure; statement of whether it is independent from the sponsor and competing interests; and reference to where further details about its charter can be found, if not in the protocol. Alternatively, an explanation of why a DMC is not needed	No data safety monitoring board has been appointed for this study as the risks of this study are assumed negligible.
Data monitoring: interim analysis	#21b	Description of any interim analyses and stopping guidelines, including who will have access to these interim results and make the final decision to terminate the trial	n/a No interim analysis will be performed
Harms	#22	Plans for collecting, assessing, reporting, and managing solicited and spontaneously reported adverse events and other unintended effects of trial interventions or trial conduct	n/a No (serious) adverse events are expected from this study

For peer review only

Auditing	#23	Frequency and procedures for auditing trial conduct, if any, and whether the process will be independent from investigators and the sponsor	N/a, no audit trial will be conducted
Research ethics approval	#24	Plans for seeking research ethics committee / institutional review board (REC / IRB) approval	12
Protocol amendments	#25	Plans for communicating important protocol modifications (eg, changes to eligibility criteria, outcomes, analyses) to relevant parties (eg, investigators, REC / IRBs, trial participants, trial registries, journals, regulators)	3, Protocol modifications will be communicated to the ethical committee, trial registry and funding body
Consent or assent	#26a	Who will obtain informed consent or assent from potential trial participants or authorised surrogates, and how (see Item 32)	11
Consent or assent: ancillary studies	#26b	Additional consent provisions for collection and use of participant data and biological specimens in ancillary studies, if applicable	10, (specific subsection in informed consent form)
Confidentiality	#27	How personal information about potential and enrolled participants will be collected, shared, and maintained in order to protect confidentiality before, during, and after the trial	8, 9
Declaration of interests	#28	Financial and other competing interests for principal investigators for the overall trial and each study site	12
Data access	#29	Statement of who will have access to the final trial dataset, and disclosure of contractual agreements that limit such access for investigators	12

For peer review only

Ancillary and post trial care	#30	Provisions, if any, for ancillary and post-trial care, and for compensation to those who suffer harm from trial participation	n/a observational study
Dissemination policy: trial results	#31a	Plans for investigators and sponsor to communicate trial results to participants, healthcare professionals, the public, and other relevant groups (eg, via publication, reporting in results databases, or other data sharing arrangements), including any publication restrictions	12
Dissemination policy: authorship	#31b	Authorship eligibility guidelines and any intended use of professional writers	13
Dissemination policy: reproducible research	#31c	Plans, if any, for granting public access to the full protocol, participant-level dataset, and statistical code	No plans for public access. Data use can be requested in order to control access and fair use.
Informed consent materials	#32	Model consent form and other related documentation given to participants and authorised surrogates	14-26 (Appendices)
Biological specimens	#33	Plans for collection, laboratory evaluation, and storage of biological specimens for genetic or molecular analysis in the current trial and for future use in ancillary studies, if applicable	10

The SPIRIT checklist is distributed under the terms of the Creative Commons Attribution License CC-BY-ND 3.0. This checklist can be completed online using https://www.goodreports.org/, a tool made by the EQUATOR Network in collaboration with Penelope.ai

For peer review only

BMJ Open

Sensitivity of point of care testing C-reactive protein and procalcitonin to diagnose urinary tract infections in Dutch nursing homes: PROGRESS study protocol

Journal:	*BMJ Open*
Manuscript ID	bmjopen-2019-031269.R1
Article Type:	Protocol
Date Submitted by the Author:	03-Jul-2019
Complete List of Authors:	Kuil, Sacha; Amsterdam UMC, Medical Microbiology;<br>Hidad, Soemeja; Amsterdam UMC, Medical Microbiology<br>Fischer, Johan; Amsterdam UMC, Clinical Chemistry<br>Harting, Janneke; Amsterdam UMC, Public Health<br>Hertogh, C; VU University Medical Center , General Practice and Elderly Care Medicine<br>Prins, Jan; Amsterdam UMC, Internal Medicine<br>van Leth, Frank; AIGHD, Global Health<br>de Jong, Menno; Amsterdam UMC, Medical Microbiology<br>Schneeberger, Caroline; Amsterdam UMC, Medical Microbiology
**Primary Subject Heading**:	Infectious diseases
Secondary Subject Heading:	Geriatric medicine, Urology
Keywords:	Point of care testing, C-Reactive protein, Procalcitonin, Nursing homes, Urinary tract infections < UROLOGY, Antimicrobial resistance

SCHOLARONE™
Manuscripts

Sensitivity of point of care testing C-reactive protein and procalcitonin to diagnose urinary tract infections in Dutch nursing homes: PROGRESS study protocol

S.D. Kuil[1#] S. Hidad[1] J.C. Fischer[2] J. Harting[3] C.M.P.M. Hertogh[4] J.M. Prins[5] F. van Leth[6, 7] M.D. de Jong[1] C. Schneeberger[1]

[1] s.d.kuil@amc.nl Amsterdam UMC, University of Amsterdam, Department of Medical Microbiology, Amsterdam Infection & Immunity Institute, Meibergdreef 9, Amsterdam, The Netherlands

[1] s.hidad@amc.nl Amsterdam UMC, University of Amsterdam, Department of Medical Microbiology, Amsterdam Infection & Immunity Institute, Meibergdreef 9, Amsterdam, The Netherlands

[2] j.c.fischer@amc.nl Amsterdam UMC, University of Amsterdam, Department of Clinical Chemistry, Meibergdreef 9, Amsterdam, The Netherlands

[3] j.harting@amc.nl Amsterdam UMC, University of Amsterdam, Department of Public Health, Amsterdam Public Health research institute, Meibergdreef 9, Amsterdam, The Netherlands

[4] cmpm.hertogh@vumc.nl Amsterdam UMC, VU University Medical Center, Department of General Practice and Elderly Care Medicine, Amsterdam Public Health, University Network of Organizations for Elderly Care, de Boelelaan 1117, Amsterdam, The Netherlands.

[5] j.m.prins@amc.nl Amsterdam UMC, University of Amsterdam, Division of Infectious Diseases, Department of Internal Medicine, Amsterdam Infection & Immunity Institute, Meibergdreef 9, Amsterdam, The Netherlands

[6] f.vanleth@aighd.org Amsterdam UMC, University of Amsterdam , Department of Global Health, Meibergdreef 9, Amsterdam, The Netherlands

[7] Amsterdam Institute for Global Health and Development, Amsterdam, The Netherlands.

[1] m.d.dejong@amc.nl Amsterdam UMC, University of Amsterdam, Department of Medical Microbiology, Amsterdam Infection & Immunity Institute, Meibergdreef 9, Amsterdam, The Netherlands

[1] c.schneeberger@amc.nl Amsterdam UMC, University of Amsterdam, Department of Medical Microbiology, Amsterdam Infection & Immunity Institute, Meibergdreef 9, Amsterdam, The Netherlands

[#] Corresponding author: Sacha D. Kuil, s.d.kuil@amc.nl, Dept. of Medical Microbiology Amsterdam UMC, University of Amsterdam, Meibergdreef 9, 1105 AZ Amsterdam, the Netherlands

Word count abstract: 295 **Word count full text:** 4243

For peer review only - http://bmjopen.bmj.com/site/about/guidelines.xhtml

ABSTRACT

Introduction:

Suspected urinary tract infection (UTI) ranks among the most common reasons for antibiotic use in nursing homes. However, diagnosing UTI in this setting is challenging because UTI often present with non-specific symptomatology. Moreover asymptomatic bacteriuria is common in elderly, which complicates attribution of causality to detection of bacteria in urine. These diagnostic challenges contribute to overuse of antibiotics and emergence of antimicrobial resistance (AMR) in nursing homes. Given the diagnostic challenges there is a need for point-of-care (POC) diagnostic tests to support clinical rules for diagnosing UTI. Procalcitonin (PCT) and C-reactive protein (CRP) are inflammatory blood markers that have been proven useful to support diagnosis and monitoring of (bacterial) respiratory tract infections and sepsis. While limited studies suggest their usefulness in supporting UTI diagnosis, their utility has not been studied in elderly populations for this purpose.

Methods and analysis:

In a 24-month matched prospective study 'PROGRESS' will assess and compare the sensitivity of rapid POC measurements of blood CRP and PCT levels to support clinical rules for diagnosing UTI in nursing home residents. The primary outcome measure is sensitivity of the POC tests to identify patients with true UTI based on the predefined definition, as derived from Receiving Operating Curves (ROC).

Ethics and dissemination:

This study will be conducted in accordance with Good Clinical Practice guidelines and the principles of the Declaration of Helsinki. The study protocol is approved by the Medical Ethical Committee (METc) of AmsterdamUMC location VUmc with reference number 2017.350 and National Central Committee on Research involving Human Subjects (CCMO) with reference number NL62067.029.17.

Registration details: Dutch trial registry: NTR6467

Keywords: Point of care testing, C-Reactive protein, Procalcitonin, Nursing homes, Urinary tract infections, Antimicrobial resistance

Strengths and limitations

Strengths

For peer review only - http://bmjopen.bmj.com/site/about/guidelines.xhtml

- Stringent post-hoc UTI criteria incorporating microbiology result and clinical response to adequate antibiotic therapy
- Sensitivity in relevant study population, namely nursing home residents

Limitations

- No on-site point of care test (POCT), however POCT is performed within 4 hours
- Single country study

Protocol version 4 March 2019, version 5

1 September 2017 Original

21 October 2017 Amendment 1: Addition of information brochures for nursing home staff and patients

20 November 2017 Amendment 2: Change of nomenclature for part of nursing home residents to temporary rehabilitation patients (at rehabilitation wards)

7 March 2018 Amendment 3: Change exclusion criterion prior inclusion to prior inclusion in the past 30 days. Subsequently to account for non-independence of observation for a limited number of participants, the sample size is adjusted with a design effect of 1.1

28 June 2018 Amendment 4: (1) Change in blood sample collection method (venipuncture to finger prick collection) due to availability of new procalcitonin POCT with small input volume, as finger prick blood sample collection is a preferred collection method in the elderly nursing home population. (2) Change sample size retaining stringent criteria (p-value 0.05 and power 90%) (3) Prolongation study duration: 12 to 18 months (4) Expansion of number of nursing homes from 11 to 12 nursing homes

3 March 2019 Amendment 5: (1) Expansion of number of nursing homes from 12 to 13 nursing homes (2) Adjusted informed consent procedure for capacitated residents

1 July 2019 Amendment 6: (1) Expansion of number of nursing homes from 13 to 14 nursing homes (2) Addition of post-hoc analysis using different UTI definitions

Introduction

Antimicrobial resistance (AMR) is mainly driven by inappropriate antibiotic use in both humans and animals. AMR is an problem worldwide. Nursing homes are increasingly regarded as an important reservoir for the emergence of AMR[1 2 3 4].

For peer review only - http://bmjopen.bmj.com/site/about/guidelines.xhtml

The most frequently reported infections in elderly residents are urinary tract infections (UTI)[5]. In Dutch nursing homes, an average weekly incidence of 10.3 (95% CI 9.8 – 10.8) per 1000 elderly residents was found[6]. UTI is the most common reason for prescribing antibiotics in Dutch nursing homes. However, not all diagnosed UTIs are “true” UTIs since recognition and diagnosing UTI in nursing homes is complex. Approximately one third of elderly residents UTIs are misdiagnosed, leading to inappropriate antibiotic use[7 8]. This is mainly due to the facts that non-specific, non-urinary tract symptoms such as altered mental status are often attributed to UTIs whilst asymptomatic bacteriuria (ASB), possibly resulting in positive urine tests, is also common in the elderly residents.[9]

Cognitive impairments and urinary tract infections

The majority of nursing homes in the Netherlands consist of psychogeriatric wards (57%), for elderly residents suffering from cognitive impairments, mainly Alzheimer disease[10 1112 13]. Their ability to verbally communicate or express classical symptoms of UTI, such as dysuria, urgency or frequency, is often limited[14]. The most frequently presented symptom in elderly residents leading to antibiotic prescription for a suspected UTI is an altered mental status (43.3%), while classical symptoms as dysuria, urgency and frequency are present in the minority of cases (0 – 3.8%)[15]. Confusion or an altered mental state are nonspecific symptoms and can result from other infectious and non-infectious diseases in the elderly residents.

Asymptomatic bacteriuria

Asymptomatic bacteriuria (ASB) is defined by the presence of significant bacteriuria without symptoms of UTI. ASB is thus regarded as colonization of the urinary tract rather than infection. ASB is highly prevalent in healthy elderly persons with reported prevalence rates as high as 40-50%[16 17]. In the presence of ASB, frequently used urine tests based on detection of bacteria are less applicable to diagnose UTI, because detection of bacteria does not discriminate between ASB and UTI. In combination with above described non-specific symptomatology, it is difficult to distinguish ASB from “true” UTI in the elderly.

For peer review only - http://bmjopen.bmj.com/site/about/guidelines.xhtml

C-reactive protein and procalcitonin

In the diagnosis of respiratory tract infections and monitoring of bacterial sepsis, inflammatory markers such as C-reactive protein (CRP) and procalcitonin (PCT) in blood have proven useful to guide antibiotic therapy and reduce antibiotic use[18 19 20 21]. Currently, point-of-care (POC) CRP measurements are recommended by Dutch guidelines for general practitioners to guide antibiotic treatment for acute respiratory tract infections. CRP and PCT represent potential candidates for rapid POC testing to support UTI diagnosis.

Ageing and frailty are associated with changes in serum inflammation protein levels, such as CRP and PCT [22 23 24]. Therefore, it is important to determine cut-off values for CRP and PCT specifically in the elderly nursing home resident population.

Inflammatory markers CRP and PCT in UTI

Studies in adults showed that CRP and PCT levels in parenchymatous infections (acute pyelonephritis, prostatitis and epididymitis) are increased[25 26]. Using a PCT-based algorithm in UTI treatment was shown to reduce antibiotic exposure in adults[27]. In a subgroup analysis including elderly (> 70 years of age) with lower UTI antibiotic exposure was reduced as well, suggesting a potential role for PCT.

In children with UTI, sensitivity of CRP (cut-off 20 mg/L) and PCT (cut-off 0.5 ng/mL) in predicting pyelonephritis is high (94% and 86% respectively), but specificity varies (39% and 74% respectively))[28]. However the number of studies in this systematic review was limited and the heterogeneity substantial. The specificity and positive predictive values varied because CRP and PCT were also increased in children with lower UTI. This suggests a possible role for inflammatory markers in distinguishing UTI (upper and lower UTI) from no inflammation (ASB), where studies are lacking.

In the elderly population only three studies have been performed evaluating CRP and PCT and their possible role in UTI[29 30 31]. These studies focused on hospitalized and more severely ill elderly populations, making the results not applicable to the nursing home population. In 13% of the healthy elderly controls CRP values were increased[30], again suggesting increased inflammation in ageing and frailty, indicating the need to determine specific cutoffs in elderly. Studies on lower UTIs and CRP or PCT in elderly are scarce.

For peer review only - http://bmjopen.bmj.com/site/about/guidelines.xhtml

Distinguishing UTI and ASB

The majority of UTIs in elderly residents are lower UTIs without fever or other signs of systemic illness[32 33] while most studies on CRP and PCT in UTI focus on diagnostic values in upper UTI or focus on distinguishing upper and lower UTI. The only small study on distinguishing lower UTI (infection) from ASB (colonization) in adults, shows a high negative predictive value (NPV) for UTI of PCT levels at a cutoff of 0.25 ng/mL[34] , suggesting that low PCT levels can rule out UTI and contribute to reducing antibiotic use.

In conclusion, decisions about who to treat and who not to treat are challenging in elderly residents with suspected UTI, resulting in potential antibiotic overuse in those who do not need treatment. Availability of a simple and rapid point-of-care (POC) test to distinguish true UTI from ASB represents an unmet need which would greatly assist clinical management of these vulnerable patients, not only by improving appropriateness of antibiotic treatment but also by enabling consideration of alternative causes of presenting non-specific symptomatology when UTI is unlikely.

Methods and analysis

Aim

To assess the sensitivity of point-of-care measurements of blood CRP and PCT levels to support clinical rules for diagnosing urinary tract infections UTI in elderly nursing home residents.

Outcome

The primary outcome is the sensitivity of the point-of-care (POC) test to identify patients with a true UTI based on the predefined definition, as derived from Receiving Operating Curves (ROC).

Design and setting

For peer review only

For peer review only - http://bmjopen.bmj.com/site/about/guidelines.xhtml

In a prospective matched study we will assess and compare the sensitivity of rapid POC measurements of blood CRP and PCT levels to support clinical rules for diagnosing UTI in elderly residents, with a post-hoc definition of UTI with stringent criteria including microbiology results as gold standard. The start date of this study is November 2017 and the planned end date is December 2019.

In this study a true UTI is present when the following five criteria are met: presence of at least two urinary or non-specific symptoms (1), positive urine leucocyte esterase test (2), presence of uropathogens in bacterial culture at $10^4 \geq$ CFU/mL (3), maximum of two uropathogens present (4) and symptom resolution in the course of adequate antibiotic treatment, where adequate treatment is defined by proven susceptibility of isolated uropathogens to the administered antibiotic (5).

The matching refers to the assessment of blood CRP and PCT levels in the same study participants. The study will be performed in nursing homes of the University Network for Organizations of Elderly Care of the VUmc University Medical Center (UNO-VUmc). The expected study duration is 24 months.
The nursing home population in this study consists mostly of psychogeriatric and somatic (long-stay) wards and some rehabilitation (short-stay) wards.

Informed consent procedure
Most nursing home patients are incapacitated. In case of incapacity legal representatives will be asked for informed consent. Capacitated patients will be asked for informed consent themselves. When nursing home staff suspect a UTI, it is not considered practical to obtain written informed consent from the representatives because preferably the blood sample should be drawn as soon as possible. Therefore informed consent will be obtained pre-emptively at the start of the study or when admitted to the nursing home. This means that patients or their legal representatives provide consent *a priori* to participate in the study once a UTI is clinically suspected during the study period. This procedure will greatly enhance feasibility of enrolment in psychogeriatric nursing home wards.

For peer review only - http://bmjopen.bmj.com/site/about/guidelines.xhtml

Inclusion and exclusion criteria

Eligible for study participation are elderly nursing home residents clinically suspected of UTI by the attending physician or nurse. Exclusion criteria are suspected respiratory tract infection, suspected other infection requiring antibiotic therapy, previous study inclusion in the past 30 days or lack of written informed consent.

Study procedures

Study enrolment

The study physician will be notified by the nursing home staff when there is potentially eligible patient and will visits the nursing home as soon as possible. The research physician will verify if eligibility criteria are met and will complete study enrolment.

Data collection

To meet our post-hoc UTI criteria data on signs and symptoms, type and duration of antibiotic used, urine leucocyte esterase, bacterial culture and antimicrobial susceptibility and clinical response are collected. Demographic, clinical and laboratory data are collected through an electronic data capture system using software of Open Data Kit[35] with which case report forms (CRFs) are designed that incorporate consistency checks to minimize incompatible data points. Data will be handled encoded, working with barcodes scanned by the database APP. Data of paper registration forms is directly entered into online CRFs and uploaded to a pre-defined database.

Data on culture results (species, susceptibility patterns by Minimal Inhibitory Concentrations MIC's) from the laboratory system will be collected in the currently used laboratory systems (Labtrain and Kiestra)

Data on signs and symptoms, clinical response and antibiotic use

The attending physician or nurse collects demographic data and data on signs and symptoms of UTI at the day of study enrolment (day 0), see Figure 1. The attending physician or nurse will evaluate improvement of signs and symptoms at day 5 and 10 and hospitalization and/or mortality at day 10. The research physician will visit the nursing homes regularly for monitoring

For peer review only - http://bmjopen.bmj.com/site/about/guidelines.xhtml

of follow-up. Data on antibiotic use (timing of initial antimicrobial therapy, type of antimicrobial agent and possible switch in antimicrobial therapy) will be collected by the research physician 10 days after study enrolment.

Urine sample collection, dipstick analysis and bacterial culture

The attending nurse will collect an urine sample for purpose of this study. Urine samples are collected spontaneously voiding either directly in a sterile urine container or in chamber pot (insert pan). When participants have an indwelling urine catheter, urine will be collected when the urine bag is changed. When participants suffer from urinary incontinence or are not able to urinate on the toilet or chamber pot, diapers will be used to obtain urine for bacterial culture and dipstick analysis [36 37] Urine extraction from diapers can provide a reliable diagnostic specimen, if fecal contamination can reasonably excluded[38]. Date, time and way of urine collection method are registered by the attending nurse. The urine sample is stored at 4°C.

For dipstick analysis Combur 2 (Roche Diagnostics) will be used (nitrite and leucocyte esterase) by the research physician. Urine will be used for semi-quantitative bacterial culture. Inoculation of 10 µL urine to CHROMID CPS Elite agar (Biomerieux) and Columbia CNA agar with 5% sheep blood (Biomerieux) will be streaked using a four quadrant pattern. Bacterial growth will be interpreted after overnight incubation at 35°C in aerobic (CPSE) and CO_2 enriched (CNA) environment. Uropathogens will be identified by Maldi-tof mass spectrometry (Microflex, Bruker Daltonic). All primary and secondary uropathogens in the European Consensus Guideline are considered as uropathogens in this study. Doubtful isolates are considered as non-uropathogens[39]. When bacterial growth of ≥10^4 CFU/mL is found, antibiotic susceptibility testing will be performed using the VITEK2 platform (BioMérieux). Unlike the European Consensus Guideline, for all uropathogens ≥ 10^4 CFU/mL growth will be used as cut-off, as urine collection methods will differ and the presence or absence of specific urinary symptoms. Participants, attending physicians and nurses will not be informed of urine dipstick and bacterial culture results (blinded).

Blood sample collection and point of care testing

For POC testing the research physician will collect a blood sample by capillary fingerprick at day 0. Capillary blood sample collection is a preferred collection method in the elderly nursing home population. For CRP testing the Afinion AS100 point-of-care (POC) platform (Alere Health B.V.,

For peer review only - http://bmjopen.bmj.com/site/about/guidelines.xhtml

Tilburg) is used. For PCT testing the Afias1 PCT Plus (Avant Medical B.V., Geffen) is used. Both, CRP and PCT platforms enable capillary blood sample collection to better facilitate future implementation in nursing homes. POC testing is performed according to the manufacturer's protocol. For logistic reasons the POC testing is performed centrally at the Medical Microbiology laboratory of our hospital. POC tests are performed within 4 hours after blood sample collection to ensure stability of CRP and PCT.

Participants, attending physicians and nurses will not be informed of POC-test results (blinded).

Biological specimen storage and molecular analysis In future

Aliquots of urine samples will be stored (-80°C) when consent is obtained specifically for genetic studies. In future studies metagenomic sequencing will be performed on bacterial DNA, to identify uropathogens and resistance genes.

Sample size

For the sample size calculation, we used a two-sided p-value of 0.05 and a power of 90%. We assume a difference in sensitivity between the two POC tests of at least 10% as clinically relevant. This difference is the net result of discordant test outcomes between the two tests. The magnitude of discordance is the main parameter in the McNemar test used to calculate the sample size for the matched design.

With a proposed sample size of 440 enrolled participants, we are able to adequately assess an increased sensitivity of 10% or more, when the prevalence of UTI in the study population is 40% or more. If the prevalence if UTI is lower, the difference in prevalence that is statistically significant (at the 5%) level increases to 11 or 12%.

Based on data of the national sentinel surveillance network for infectious diseases in nursing homes (SNIV) in 2015, the prevalence of UTI in Dutch nursing homes is 10.3 in 1000 patient weeks. UTI definition by SNIV is broader where response to adequate antibiotic treatment is not taken into account.

In order to reach the target, the PROGRESS study will take place in 13 nursing homes with around 1350 elderly residents in total. Recruitment of a manageable number of approximately 12 participants per week in total is anticipated.

For peer review only - http://bmjopen.bmj.com/site/about/guidelines.xhtml

Data and statistical analysis

The sensitivity of both POC tests to diagnose UTIs defined by the post-hoc definition is derived from the optimal point of a Receiver Operating Curve (ROC). Sensitivities of CRP and PCT tests are compared by a matched analysis approach using a two-tailed McNemar test assessing the statistical significance of differences between the sensitivities of each pair of tests (performed in each study participant).

Patient and public involvement

Patients or public are not involved in designing this study.

This study protocol is written using the SPIRIT reporting guidelines[40]

Ethics and dissemination:

This study will be conducted in accordance with Good Clinical Practice guidelines and the principles of the Declaration of Helsinki. The study protocol is approved by the Medical Ethical Committee (METc) of AmsterdamUMC location VUmc with reference number 2017.350 and National Central Committee on Research involving Human Subjects (CCMO) with reference number NL62067.029.17. Written informed consent from nursing home residents or legal representatives (when incapacitated) will be obtained prior to study enrolment. Informed consent from residents will be obtained directly by researchers or via mail. Informed consent from legal representatives will be obtained via mail. Treating physicians decide which residents are capacitated.

Data safety monitoring board

No data safety monitoring board has been appointed for this study as the risks of this study are assumed negligible.

Discussion

This trial will test the hypothesis that increased levels of inflammatory markers support the

For peer review only - http://bmjopen.bmj.com/site/about/guidelines.xhtml

diagnosis of UTI in elderly and can guide empirical antibiotic treatment in nursing homes. Although our study involves the target population (elderly nursing home residents), found CRP and/or PCT cut-off values need to be confirmed by prospective trials. Besides test sensitivity the potential impact on antibiotic prescription needs to be established in a consecutive, randomized controlled, study[41]. The aim of this study is assessing the sensitivity of POCT CRP and PCT in current clinical practice. This clinical setting suboptimal for urine collection, where urine incontinence complicates collection methods. Chamber pots and diapers are frequently used, which introduces the risk of UTI overestimation by positive dipstick urinalysis and bacterial cultures. Although we have shown in our add-on laboratory study that diapers can be used for UTI diagnosis, the outcome can be affected by suboptimal urine collection.

In this population a reference test for UTIs does not exist, this is the actual gap we are trying to address. To reduce classification bias when a reference test is lacking we use a post-hoc definition of 'true' UTI[42]. We defined a stringent definition of UTI that in our opinion makes a clear distinction between UTI and ASB. We will perform post-hoc analysis in which UTI definition is less stringent to assess the effects of potential misclassification of the outcome. UTI definition in this post-hoc analysis will include classical UTI symptoms irrespective of resolution with adequate antibiotics, in accordance with the Dutch national guidelines[43]. We will include this procedure in the current amendment and data analysis plan that is drawn up before the completion of the data collection.

Since effectiveness of a new test does not just depend on its proven efficacy in research studies, but also on successful implementation after the study, qualitative research on identifying the barriers and facilitators for implementation are needed and will be performed in parallel to the described study.

Figure legend

Figure 1 Data collection PROGRESS study

* Participants and attending physicians/nurses will not be informed of results (blinded)

POCT – Point of Care Testing; CRP – C-reactive protein; PCT – Procalcitonin

List of abbreviations

Amsterdam UMC Amsterdam Universitair Medische Centra

AMR Antimicrobial resistance

For peer review only

For peer review only - http://bmjopen.bmj.com/site/about/guidelines.xhtml

ASB Asymptomatic bacteriuria
CCMO National Central Committee on Research involving Human Subjects
CFU Colony forming units
CRP C-reactive protein

MIC Minimal inhibitory concentration
NPV Negative predictive value
ODK Open data kit
PCT Procalcitonin
POC Point of care
SNIV Surveillance network for infectious diseases in nursing homes
UNO Universitair netwerk ouderenzorg
UTI urinary tract infection
VUmc Vrije Universiteit medisch centrum

Declarations

Dissemination policy

According to the CCMO statement on publication policy, the results of this study will be disclosed unreservedly.

Competing interests

No competing interests.

Funding

This study is funded by The Netherlands Organization for Health Research and Development (ZonMW) grant 541001003. ZonMW, Laan van Nieuw Oost Indië 334, 2593 CE Den Haag, The Netherlands. The study protocol is being peer reviewed by the funder. The funder has no role in collection, management, analysis, and interpretation of data, writing of the report or the decision to submit the report for publication.

Roles and responsibilities

Research physician/PhD student (SDK): coordinating PROGRESS, patient recruiting, data collection, specimen handling, POC and urine testing, preparation protocols, CRFs and

For peer review only

For peer review only - http://bmjopen.bmj.com/site/about/guidelines.xhtml

publication of study reports including annual ethical committee report.
Research assistant (SH): patient recruiting, data collection, specimen handling, POC and urine testing
Principal investigator (MdJ), senior researcher (CS) and lead epidemiologist (FvL): Study planning, agreement of final protocols, reviewing progress of study
Lead epidemiologist (FvL): design of the electronic report form (eCRF), database design and maintenance, data validation and data management plans, user account maintenance, design of automated data validation scripts, preparation of the final data anlysis sets, supervising of data analysis.
Laboratory chemist (JCF): responsible for verification of POCT, contractual issues with manufacturers
In each participating center a lead investigator (elderly care physician) will be identified, to be responsible for progress monitoring and assisting with study set-up per site.

Author contributions

SDK recruits patients, coordinates and performs the clinical study and the wrote the manuscript. SH recruits patients and performs the clinical study. FvL, JCF, JH, CMPMH, JMP, MDdJ and CS developed the protocol and secured funding for this project. CS, FvL and MdJ supervised the design of the study and writing this manuscript. FvL provided the statistical analysis plan, database set-up. All authors have read and approved the manuscript.

For peer review only

For peer review only - http://bmjopen.bmj.com/site/about/guidelines.xhtml

References

[1] Rooney PJ, O'Leary MC, Loughrey AC, McCalmont M, Smyth B, Donaghy P, Badri M, Woodford N, Karisik E, Livermoore DM Nursing homes as a reservoir of extended-spectrum beta-lactamase (ESBL)-producing ciprofloxacin-resistant Escherichia coli. J Antimicrob Chemother. 2009;64(3):635-41.
[2] Lee BY, Bartsch SM, Wong KF, Singh A, Avery TL, Kim DS, Brown ST, Murphey CR, Yilmaz SL, Potter MA, Huang SS. The importance of nursing homes in the spread of methicillin-resistant Staphylococcus aureus (MRSA) among hospitals. Med Care. 2013;51(3):205-15.
[3] Kahvecioglu D, Ramiah K, McMaughan D, Garfinkel S, McSorley VE, Nguyen QN, Yang M, Pugliese C, Mehr D, Philips CD. Multidrug-resistant organism infections in US nursing homes: a national study of prevalence, onset, and transmission across care settings, October 1, 2010-December 31, 2011. Infect Control Hosp Epidemiol. 2014;35 Suppl 3:S48-55.
[4] Bedenic B, Beader N, Godic-Torkar K, Vranić-Ladavac M, Luxner J, Veir Z, Grisold AJ, Zarfel G. Nursing Home as a Reservoir of Carbapenem-Resistant Acinetobacter baumannii. Microb Drug Resist. 2015;21(3):270-8.
[5] Lutters M, Vogt-Ferrier NB. Antibiotic duration for treating uncomplicated, symptomatic lower urinary tract infections in elderly women. Cochrane Database of Systematic Reviews. 2008; doi:10.1002/14651858.CD001535.pub2
[6] Rijksinstituut voor Volksgezondheid en Milieu. Surveilance Netwerk Infectieziekten in Verpleeghuizen. Resultaten van wekelijkse surveillance. Referentiecijfers 2011 – 2015 https://www.rivm.nl/sites/default/files/2018-11/Referentiecijfers%20Incidentie%20SNIV%202011-2015_def.pdf Accessed 7 March 2019
[7] van Buul LW, Veenhuizen RB, Achterberg WP, Schellevis FG, Essink RT, de Greeff SC, Natsch S, van der Steen JT, Hertogh CM. Antibiotic Prescribing In Dutch Nursing Homes: How Appropriate Is It? J Am Med Dir Assoc. 2015; 16(3):229-37
[8] Loeb M, Simor AE, Landry L, et al. Antibiotic use in Ontario facilities that provide chronic care. J Gen Intern Med. 2001;16:376–383.
[9] van Buul LW, Vreeken HL, Bradley SF, Crnich CJ, Drinka PJ, Geerlings SE, Jump RLP, Mody L, Mylotte JJ, Loeb M, Nace DA, Nicolle LE, Sloane PD, Stuart RL, Sundvall PD, Ulleryd P, Veenhuizen RB, Hertogh CMPM. The Development of a Decision Tool for the Empiric Treatment of Suspected Urinary Tract Infection in Frail Older Adults: A Delphi Consensus Procedure. J Am Med Dir Assoc. 2018. 19(9):757-764
[10] Schols, J and Kardol T. Dementia care in nursing homes requires a multidisciplinary approach. In: Schüssler S and Lohrmann C. Dementia in nursing homes. 2017. Chapter 15, p. 210.
[11] Alzheimer's disease facts and figures. Alzheimer's Association. 2017 Alzheimer's & Dementia 2017;13(4):325-373
[12] Shah DC, Evans M, King D. Prevalence of mental illness in a rehabilitation unit for older adults. Postgrad Med J. 2000; 76(893):153-6.
[13] Heeren THJ, Lagaay AM, Rooijmans HGM. De prevalentie van het dementiesyndroom bij de oudste bewoners van het somatisch verpleeghuis. Ned Tijdsch Geneeskd 1992;136(14):695-698
[14] Sobel JD and Kaye D. Urinary tract infections. In: Mandell, Douglas and Bennett. Principles and practice of infectious diseases 8th edition, Chapter 74, p.896
[15] D'Agata, E, Loeb MB, and Mitchell SL. Challenges Assessing Nursing Home Residents with Advanced Dementia for Suspected Urinary Tract Infections. J Am Geriatr Soc. 2013;61(1):62–66
[16] Hedin K, Petersson C, Widebäck K, Kahlmeter G, Mölstad S. Asymptomatic bacteriuria in a population of elderly in municipal institution care. Scand J Prim Health Care. 2002;20(3):166-8
[17] Eberle CM, Winsemius D, Garibaldi RA. Risk factors and consequences of bacteriuria in non catheterized nursing home residents. J Geront 1993;48(6):M266-71
[18] Do NT, Ta NT, Tran NT, et al. Point-of-care C-reactive protein testing to reduce inappropriate use of antibiotics for non-severe acute respiratory infections in Vietnamese primary health care: a randomised controlled trial. Lancet Glob Health. 2016;4(9):e633-41
[19] Agency for Health Care Research and Quality. Procalcitonine-guided antibiotic therapy. Comparative Effectiveness Review. 2012; (78):1-16

For peer review only - http://bmjopen.bmj.com/site/about/guidelines.xhtml

https://effectivehealthcare.ahrq.gov/sites/default/files/related_files/procalcitonin_executive.pdf Accessed 7 March 2019
[20] Aabenhus R, Jensen JUS, Jørgensen KJ uhl, Hróbjartsson A, Bjerrum L. Biomarkers as point-of-care tests to guide prescription of antibiotics in patients with acute respiratory infections in primary care. Cochrane database Syst Rev. 2014;11. doi:10.1002/14651858.CD010130.pub2.
[21] Schuetz P, Wirz Y, Sager R, et al. Procalcitonin to initiate or discontinue antibiotics in acuterespiratory tract infections (Review). Cochrane Database Syst Rev. 2017;Art. No.:(10). doi:10.1002/14651858.CD007498.pub3.www.cochranelibrary.com.
[22] Soysal P, Stubbs B, Lucato P, Luchini C, Solmi M, Peluso R, Sergi G, Isik AT, Manzato E, Maggi S, Maggio M, Prina AM, Cosco TD, Wu YT, Veronese N. Inflammation and frailty in the elderly: A systematic review and meta-analysis. Ageing Research Reviews. 2016;(31):1-8
[23] Chenevier-Gobeaux C, Trabattoni E, Elfassy Y, Picard C, Guérin S, Borderie D, Claessens YE. Decisional procalcitonin thresholds are not adapted to elderly patients admitted to the emergency room. Biomarkers. 2012;17(5):477-481.
[24] Woloshin S, Schwartz LM Distribution of C-reactive protein values in the United States. N Engl J Med 2005;352(15):1611-3.
[25] Sugimoto K, Shimizu N, Matsumura N, Oki T, Nose K, Nishioka T, Uemura H. Procalcitonin as a useful marker to decide upon intervention for urinary tract infection. Infect Drug Resist. 2013;7(6):83-6.
[26] Chuang YC, Vikas T, Liu RT, Chancellor MB, Tyagi P. Urine and Serum C-Reactive Protein Levels as Potential Biomarkers of Lower Urinary Tract Symptoms. Urol Sci 2010;21(3):132-136.
[27] Drozdov D, Schwarz S, Kutz A, Grolimund E, Rast AC, Steiner D, Regez K, Schild U, Guglielmetti M, Conca A, Reutlinger B, Ottinger C, Buchkremer F, Haubitz S, Blum, C, Huber A, Buergi U, Schuetz P, Bock A, Fux CA, Mueller B, Albrich WC. Procalcitonin and pyuria-based algorithm reduces antibiotic use in urinary tract infections: a randomized controlled trial. BMC Med. 2015;1;13:104.
[28] Shaikh N, Borrell JL, Evron J, Leeflang MMG. Procalcitonin, C-reactive protein, and erythrocyte sedimentation rate for the diagnosis of acute pyelonephritis in children. Cochrane database Syst Rev. 2015;(1):CD009185. doi:10.1002/14651858.CD009185.pub2.
[29] Lai CC, Chen SY, Wang CY, Wang JY, Su CP, Liao CH, Tan CK, Huang YT, Lin HI, Hsueh PR. Diagnostic Value of Procalcitonin for Bacterial Infection in Elderly Patients in the Emergency Department J Am Geriatr Soc. 2010;58(3):518-22
[30] Dwolatzky T, Olshtain-Pops K, Yinnon AM, Raveh D, Rogowski O, Shapira I, Rotstein R, Berliner S, Rudensky B. Procalcitonin in the elderly: normal plasma concentrations and response to bacterial infections. Eur J Clin Microbiol Infect Dis. 2005;24(11):763-5.
[31] Liu A, Bui T, van Nguyen H, Ong B, Shen Q, Kamalasena D. Serum C-reactive protein as a biomarker for early detection of bacterial infection in the older Patient. Age and Ageing 2010; 39: 559–565.
[32] Johansen TE, Botto H, Cek M, Grabe M, Tenke P, Wagenlehner FM, Naber KG. Critical review of current definitions of urinary tract infections and proposal of an EAU/ESIU classification system. Int J Antimicrob Agents. 2011;38 Suppl:64-70
[33] Foxman B. Urinary tract infection syndromes: occurrence, recurrence, bacteriology, risk factors, and disease burden. Infect Dis Clin North Am. 2014; 28(1):1-13.
[34] Levine AR, Tran M, Shepherd J, Naut E. Utility of initial procalcitonin values to predict urinary tract infection. American Journal of Emergency Medicine. 2018; 36(11):1993-1997.
[35] Open Data Kit http://www.opendatakit.org/ Accessed 7 March 2019
[36] Midthun SJ, Paur RA, Lindseth G, Von Duvillard SP.Bacteriuria detection with a urine dipstick applied to incontinence pads of nursing home residents. Geriatr Nurs. 2003;24(4):206-9
[37] Belmin J, Hervias Y, Avellano E, Oudart O, Durand I. Reliability of sampling urine from disposable diapers in elderly incontinent women. J Am Geriatr Soc. 1993;41(11):1182-6
[38] Diaper urine: a reliable alternative for obtaining urine samples for UTI diagnosis in elderly suffering from urine incontinence. Poster session P2137, 24 April 2018 Session: Urinary tract infection - risks, diagnosis, treatment. ECCMID 2018 Madrid, Spain. https://www.escmid.org/escmid_publications/escmid_elibrary/ Accessed 7 March 2019
[39] Aspevall O, Hallander H, Gant V, Kouri T. European guidelines for urinalysis: a collaborative document produced by European clinical microbiologists and clinical chemists under ECLM in collaboration with ESCMID. Scand J Clin Lab Invest. 2000;60:1-96.

For peer review only - http://bmjopen.bmj.com/site/about/guidelines.xhtml

[40] Chan A-W, Tetzlaff JM, Altman DG, Laupacis A, Gøtzsche PC, Krleža-Jerić K, Hróbjartsson A, Mann H, Dickersin K, Berlin J, Doré C, Parulekar W, Summerskill W, Groves T, Schulz K, Sox H, Rockhold FW, Rennie D, Moher D. SPIRIT 2013 Statement: Defining standard protocol items for clinical trials. Ann Intern Med. 2013;158(3):200-207
[41] Bossuyt PMM, Reitsma JB, Linnet K, Moons KGM. Beyond Diagnostic Accuracy : The Clinical Utility of Diagnostic Tests. Clin Chem. 2012;58(12):1636-1643. doi:10.1373/clinchem.2012.182576.
[42] Reitsma JB, Rutjes AWS, Khan KS, Coomarasamy A, Bossuyt PM. A review of solutions for diagnostic accuracy studies with an imperfect or missing reference standard. Journal of Clinical Epidemiology. 2009; 62:797e806

[43] Verenso. Richtlijn Urineweginfecties bij kwetsbare ouderen. 2018. Dutch national guideline urinary tract infections in eldelry.

For peer review only

For peer review only - http://bmjopen.bmj.com/site/about/guidelines.xhtml

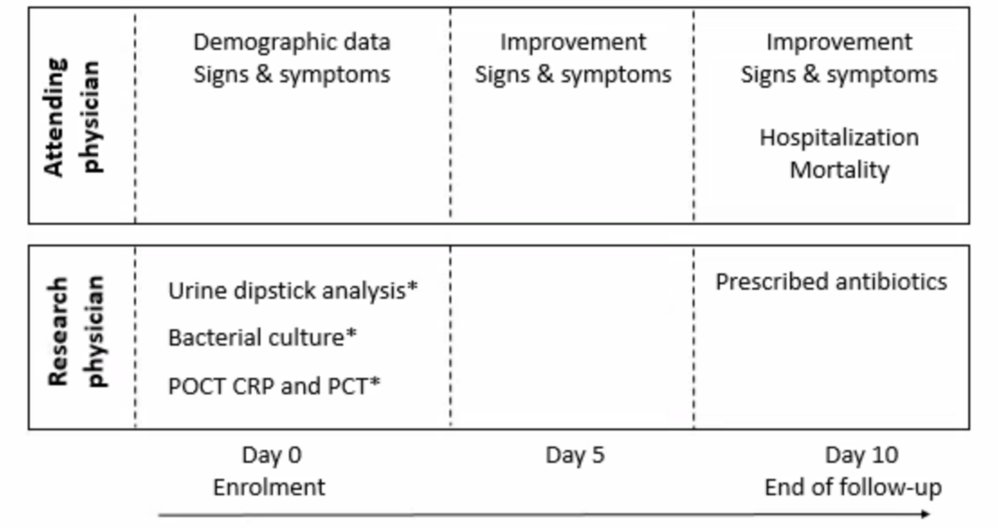

Figure 1 Data collection PROGRESS study
* Participants and attending physicians/nurses will not be informed of results (blinded)
POCT – Point of Care Testing; CRP – C-reactive protein; PCT – Procalcitonin

Reporting checklist for protocol of a clinical trial.

Based on the SPIRIT guidelines.

Instructions to authors

Complete this checklist by entering the page numbers from your manuscript where readers will find each of the items listed below.

Your article may not currently address all the items on the checklist. Please modify your text to include the missing information. If you are certain that an item does not apply, please write "n/a" and provide a short explanation.

Upload your completed checklist as an extra file when you submit to a journal.

In your methods section, say that you used the SPIRIT reporting guidelines, and cite them as:

Chan A-W, Tetzlaff JM, Altman DG, Laupacis A, Gøtzsche PC, Krleža-Jerić K, Hróbjartsson A, Mann H, Dickersin K, Berlin J, Doré C, Parulekar W, Summerskill W, Groves T, Schulz K, Sox H, Rockhold FW, Rennie D, Moher D. SPIRIT 2013 Statement: Defining standard protocol items for clinical trials. Ann Intern Med. 2013;158(3):200-207

		Reporting Item	Page Number
Title	#1	Descriptive title identifying the study design, population, interventions, and, if applicable, trial acronym	1
Trial registration	#2a	Trial identifier and registry name. If not yet registered, name of intended registry	2
Trial registration: data set	#2b	All items from the World Health Organization Trial Registration Data Set https://www.who.int/ictrp/network/trds_1.2.1/en/	First enrollment: 23-11-2017. Recruitment status: recruiting<br><br>Other items in manuscript
Protocol version	#3	Date and version identifier	3
Funding	#4	Sources and types of financial, material, and other support	12

For peer review only

Roles and responsibilities: contributorship	#5a	Names, affiliations, and roles of protocol contributors	13
Roles and responsibilities: sponsor contact information	#5b	Name and contact information for the trial sponsor<br><br>Contact information medical microbiologie AmsterdamUMC: Prof. Dr. M.D. de Jong University of Amsterdam, Department of Medical Microbiology, Amsterdam Infection & Immunity Institute, Meibergdreef 9, Amsterdam, The Netherlands	12
Roles and responsibilities: sponsor and funder	#5c	Role of study sponsor and funders, if any, in study design; collection, management, analysis, and interpretation of data; writing of the report; and the decision to submit the report for publication, including whether they will have ultimate authority over any of these activities	12, 13
Roles and responsibilities: committees	#5d	Composition, roles, and responsibilities of the coordinating centre, steering committee, endpoint adjudication committee, data management team, and other individuals or groups overseeing the trial, if applicable (see Item 21a for data monitoring committee)	13
Background and rationale	#6a	Description of research question and justification for undertaking the trial, including summary of relevant studies (published and unpublished) examining benefits and harms for each intervention	4-6
Background and rationale: choice of comparators	#6b	Explanation for choice of comparators	4-6
Objectives	#7	Specific objectives or hypotheses	6
Trial design	#8	Description of trial design including type of trial (eg, parallel group, crossover, factorial, single group), allocation ratio, and framework (eg, superiority, equivalence, non-inferiority, exploratory)	6, 7

For peer review only

Study setting	#9	Description of study settings (eg, community clinic, academic hospital) and list of countries where data will be collected. Reference to where list of study sites can be obtained	6, 7
Eligibility criteria	#10	Inclusion and exclusion criteria for participants. If applicable, eligibility criteria for study centres and individuals who will perform the interventions (eg, surgeons, psychotherapists)	8
Interventions: description	#11a	Interventions for each group with sufficient detail to allow replication, including how and when they will be administered	n/a observational study
Interventions: modifications	#11b	Criteria for discontinuing or modifying allocated interventions for a given trial participant (eg, drug dose change in response to harms, participant request, or improving / worsening disease)	n/a observational study
Interventions: adherance	#11c	Strategies to improve adherence to intervention protocols, and any procedures for monitoring adherence (eg, drug tablet return; laboratory tests)	n/a observational study
Interventions: concomitant care	#11d	Relevant concomitant care and interventions that are permitted or prohibited during the trial	n/a
Outcomes	#12	Primary, secondary, and other outcomes, including the specific measurement variable (eg, systolic blood pressure), analysis metric (eg, change from baseline, final value, time to event), method of aggregation (eg, median, proportion), and time point for each outcome. Explanation of the clinical relevance of chosen efficacy and harm outcomes is strongly recommended	6
Participant timeline	#13	Time schedule of enrolment, interventions (including any run-ins and washouts), assessments, and visits for participants. A schematic diagram is highly recommended (see Figure)	8, 9
Sample size	#14	Estimated number of participants needed to achieve study objectives and how it was determined, including	10

For peer review only

		clinical and statistical assumptions supporting any sample size calculations	
Recruitment	#15	Strategies for achieving adequate participant enrolment to reach target sample size	10
Allocation: sequence generation	#16a	Method of generating the allocation sequence (eg, computer-generated random numbers), and list of any factors for stratification. To reduce predictability of a random sequence, details of any planned restriction (eg, blocking) should be provided in a separate document that is unavailable to those who enrol participants or assign interventions	n/a observational study
Allocation concealment mechanism	#16b	Mechanism of implementing the allocation sequence (eg, central telephone; sequentially numbered, opaque, sealed envelopes), describing any steps to conceal the sequence until interventions are assigned	n/a observational study
Allocation: implementation	#16c	Who will generate the allocation sequence, who will enrol participants, and who will assign participants to interventions	n/a
Blinding (masking)	#17a	Who will be blinded after assignment to interventions (eg, trial participants, care providers, outcome assessors, data analysts), and how	n/a observational study
Blinding (masking): emergency unblinding	#17b	If blinded, circumstances under which unblinding is permissible, and procedure for revealing a participant's allocated intervention during the trial	n/a observational study
Data collection plan	#18a	Plans for assessment and collection of outcome, baseline, and other trial data, including any related processes to promote data quality (eg, duplicate measurements, training of assessors) and a description of study instruments (eg, questionnaires, laboratory tests) along with their reliability and validity, if known. Reference to where data collection forms can be found, if not in the protocol	8
Data collection plan: retention	#18b	Plans to promote participant retention and complete follow-up, including list of any outcome data to be	8

For peer review only

		collected for participants who discontinue or deviate from intervention protocols	
Data management	#19	Plans for data entry, coding, security, and storage, including any related processes to promote data quality (eg, double data entry; range checks for data values). Reference to where details of data management procedures can be found, if not in the protocol	8
Statistics: outcomes	#20a	Statistical methods for analysing primary and secondary outcomes. Reference to where other details of the statistical analysis plan can be found, if not in the protocol	10
Statistics: additional analyses	#20b	Methods for any additional analyses (eg, subgroup and adjusted analyses)	n/a
Statistics: analysis population and missing data	#20c	Definition of analysis population relating to protocol non-adherence (eg, as randomised analysis), and any statistical methods to handle missing data (eg, multiple imputation)	10
Data monitoring: formal committee	#21a	Composition of data monitoring committee (DMC); summary of its role and reporting structure; statement of whether it is independent from the sponsor and competing interests; and reference to where further details about its charter can be found, if not in the protocol. Alternatively, an explanation of why a DMC is not needed	No data safety monitoring board has been appointed for this study as the risks of this study are assumed negligible.
Data monitoring: interim analysis	#21b	Description of any interim analyses and stopping guidelines, including who will have access to these interim results and make the final decision to terminate the trial	n/a No interim analysis will be performed
Harms	#22	Plans for collecting, assessing, reporting, and managing solicited and spontaneously reported adverse events and other unintended effects of trial interventions or trial conduct	n/a No (serious) adverse events are expected from this study

For peer review only

Auditing	#23	Frequency and procedures for auditing trial conduct, if any, and whether the process will be independent from investigators and the sponsor	N/a, no audit trial will be conducted
Research ethics approval	#24	Plans for seeking research ethics committee / institutional review board (REC / IRB) approval	12
Protocol amendments	#25	Plans for communicating important protocol modifications (eg, changes to eligibility criteria, outcomes, analyses) to relevant parties (eg, investigators, REC / IRBs, trial participants, trial registries, journals, regulators)	3, Protocol modifications will be communicated to the ethical committee, trial registry and funding body
Consent or assent	#26a	Who will obtain informed consent or assent from potential trial participants or authorised surrogates, and how (see Item 32)	11
Consent or assent: ancillary studies	#26b	Additional consent provisions for collection and use of participant data and biological specimens in ancillary studies, if applicable	10, (specific subsection in informed consent form)
Confidentiality	#27	How personal information about potential and enrolled participants will be collected, shared, and maintained in order to protect confidentiality before, during, and after the trial	8, 9
Declaration of interests	#28	Financial and other competing interests for principal investigators for the overall trial and each study site	12
Data access	#29	Statement of who will have access to the final trial dataset, and disclosure of contractual agreements that limit such access for investigators	12

For peer review only

Ancillary and post trial care	#30	Provisions, if any, for ancillary and post-trial care, and for compensation to those who suffer harm from trial participation	n/a observational study
Dissemination policy: trial results	#31a	Plans for investigators and sponsor to communicate trial results to participants, healthcare professionals, the public, and other relevant groups (eg, via publication, reporting in results databases, or other data sharing arrangements), including any publication restrictions	12
Dissemination policy: authorship	#31b	Authorship eligibility guidelines and any intended use of professional writers	13
Dissemination policy: reproducible research	#31c	Plans, if any, for granting public access to the full protocol, participant-level dataset, and statistical code	No plans for public access. Data use can be requested in order to control access and fair use.
Informed consent materials	#32	Model consent form and other related documentation given to participants and authorised surrogates	14-26 (Appendices)
Biological specimens	#33	Plans for collection, laboratory evaluation, and storage of biological specimens for genetic or molecular analysis in the current trial and for future use in ancillary studies, if applicable	10

The SPIRIT checklist is distributed under the terms of the Creative Commons Attribution License CC-BY-ND 3.0. This checklist can be completed online using https://www.goodreports.org/, a tool made by the EQUATOR Network in collaboration with Penelope.ai

For peer review only
